# Vasculature analysis of patient derived tumor xenografts using species-specific PCR assays: evidence of tumor endothelial cells and atypical VEGFA-VEGFR1/2 signalings

**DOI:** 10.1186/1471-2407-14-178

**Published:** 2014-03-13

**Authors:** Ivan Bieche, Sophie Vacher, David Vallerand, Sophie Richon, Rana Hatem, Ludmilla De Plater, Ahmed Dahmani, Fariba Némati, Eric Angevin, Elisabetta Marangoni, Sergio Roman-Roman, Didier Decaudin, Virginie Dangles-Marie

**Affiliations:** 1 Laboratoire d’Oncogénétique, 35 rue Dailly, Institut Curie - Hôpital Rene Huguenin, St Cloud, France; 2 INSERM UMR745, Sorbonne Paris Cité, 4 avenue de l’Observatoire, Paris, France; 3 Département de Recherche Translationnelle, Laboratoire d’Investigation Préclinique, Paris, France; 4 Roche SAS, 30, cours de l'Ile Seguin, 92650 Boulogne-Billancourt, Cedex, France; 5 IFR71, Sorbonne Paris Cité, 4 avenue de l’Observatoire, Paris, France; 6 CNRS, UMR 144, Centre de Recherche, Institut Curie, 26 rue d’Ulm, Paris, France; 7 Institut de Cancérologie Gustave Roussy, 39 rue Camille Desmoulins, Villejuif, France; 8 Département d’Oncologie Médicale, Institut Curie, 26 rue d’Ulm, Paris, France; 9 Université Paris Descartes, Sorbonne Paris Cité, 4 avenue de l’Observatoire, Paris, France; 10 Research Center, Institut Curie, 12 rue Lhomond, F-75005 Paris, France

**Keywords:** Tumor vasculature, Patient-derived xenografts, Species-specific PCR assays, Endothelial markers, VEGFA-VEGFR1/2 signalings

## Abstract

**Background:**

Tumor endothelial transdifferentiation and VEGFR1/2 expression by cancer cells have been reported in glioblastoma but remain poorly documented for many other cancer types.

**Methods:**

To characterize vasculature of patient-derived tumor xenografts (PDXs), largely used in preclinical anti-angiogenic assays, we designed here species-specific real-time quantitative RT-PCR assays. Human and mouse *PECAM1/CD31, ENG/CD105, FLT1/VEGFR1, KDR*/*VEGFR2* and *VEGFA* transcripts were analyzed in a large series of 150 PDXs established from 8 different tumor types (53 colorectal, 14 ovarian, 39 breast and 15 renal cell cancers, 6 small cell and 5 non small cell lung carcinomas, 13 cutaneous melanomas and 5 glioblastomas) and in two bevacizumab-treated non small cell lung carcinomas xenografts.

**Results:**

As expected, mouse cell proportion in PDXs -evaluated by quantifying expression of the housekeeping gene *TBP-* correlated with all mouse endothelial markers and human *VEGFA* RNA levels. More interestingly, we observed human *PECAM1/CD31 and ENG/CD105* expression in all tumor types, with higher rate in glioblastoma and renal cancer xenografts. Human *VEGFR* expression profile varied widely depending on tumor types with particularly high levels of human *FLT1*/*VEGFR1* transcripts in colon cancers and non small cell lung carcinomas, and upper levels of human *KDR*/*VEGFR2* transcripts in non small cell lung carcinomas. Bevacizumab treatment induced significant low expression of mouse *Pecam1/Cd31, Eng/Cd105*, *Flt1*/*Vegfr1* and *Kdr*/*Vefr2* while the human *PECAM1/CD31* and *VEGFA* were upregulated.

**Conclusions:**

Taken together, our results strongly suggest existence of human tumor endothelial cells in all tumor types tested and of both stromal and tumoral autocrine VEGFA-VEGFR1/2 signalings. These findings should be considered when evaluating molecular mechanisms of preclinical response and resistance to tumor anti-angiogenic strategies.

## Background

Tumor vasculature, a crucial feature in cancer development and progression, is based on angiogenesis and vasculogenesis driven by VEGF signalings [1-3] but also on tumor endothelial transdifferentiation and vascular mimicry [4]. The VEGFR1 and VEGFR2 tyrosine kinase receptors are primarily expressed by endothelial cells. Recent studies, however, suggest that tumor-derived VEGF provides not only paracrine survival cues for endothelial cells, but may also autocrine processes in tumor cells expressing VEGFRs and play a role in tumor resistance to existing anti-angiogenic therapies [5-7].

Growth of patient tumor fragments into immunodeficient mice allows an accurate depiction of human tumor biological characteristics and are considered to represent the heterogeneity of human cancers (for review [8]). These patient-derived tumor xenografts (PDX) are greatly helpful to evaluate fundamental issues in cancer and chemosensitivity response, including characteristics of angiogenesis, tumor-stroma interactions and response to antiangiogenic therapies. As real-time quantitative RT-PCR is highly specific, species-specific primer sets can allow to discriminating between mouse/stromal and human/cancer gene expression in PDX models.

To obtain further insight into tumor vascularization and VEGFR expression by cancer and non-tumor cells, we used real-time qRT-PCR to quantify species-specific mRNAs of *PECAM1/CD31, ENG/CD105, FLT1/VEGFR1, KDR/VEGFR2* and *VEGFA* genes in a large series of 150 xenografts from different tumor types. We also validated clinical relevance of species-specific PCR assays for *in vivo* evaluation of anti-angiogenesis therapy in two non small cell lung carcinoma models. We showed human *PECAM1/CD31 and ENG/CD105* expression in all tumor types, supporting existence of human tumor endothelial cells in all tumor types. In addition, the *VEGFR* expression profiles led to involvement of both stromal and tumoral autocrine VEGFA-VEGFR1/2 signalings in tumors.

## Results and discussion

First, the proportion of mouse cells was estimated in a panel of 8 different PDX types, using a real-time qRT-PCR assay combining primers specific for mouse *Tbp* RNA and primers able to amplify a common sequence on both human and mouse *TBP* transcripts. (Additional file 1: Table S1). As this gene encoding the TATA box-binding protein is a robust house-keeping gene [9] with similar amplification efficiency for the 2 primer sets, the ratio reflects the percentage of mouse cells within xenograft as validated in a standard curve of mouse and human cDNA mixtures (data not shown).

In an initial series of 157 human xenografts, the proportion of mouse cells was 100% in 7 tumors. These 7 tumor samples probably originated from spontaneous mouse lymphoma, frequently observed in immunodeficient mice [10].

In the 150 other xenografts, mouse host cells were found in all specimens with a median proportion of mouse cells of 9%, ranged between 3.3% in SCLC and 20% in NSCLC (*p* < 0.05, Table 1). To note, all the xenografts used here, have been passaged at least 5 times in mice, leading to a replacement of human stroma by mouse components [8].

**Table 1:** Normalized gene expression for each of the 150 PDX samples, classified by tumor type (noted in bold)

**Sample nature**	**Derived from primary tumor or metastatis**	**% of mouse cells**	**PECAM1**		**ENG**		**VEGFR1**		**VEGFR2**		**VEGFA**		**% of *mVegfa *** **vs human + mouse VEGFA transcripts**
			**Hs**	**Mm**	**Hs**	**Mm**	**Hs**	**Mm**	**Hs**	**Mm**	**Hs**	**Mm**	
Pure human control		0%	1265	0	796	0	2610	0	157	0	287	0	
Pure mouse control		100%	0	1176	0	736	0	303	0	879	0	790	
**Colorectal carcinoma PDX**													
CRC#1	Primary	11%	0	894	2	492	23	453	0	405	4010	212	5%
CRC#2	Primary	5%	0	917	3	398	9	383	0	309	4912	51	1%
CRC#3	Metastasis	21%	1	2380	34	893	14	843	0	803	4642	628	12%
CRC#4	Primary	17%	0	836	<1	285	0	368	0	299	2876	302	10%
CRC#5	Metastasis	8%	0	813	0	492	3	337	0	374	3552	109	3%
CRC#6	Primary	9%	46	458	217	326	77	196	<1	176	1866	84	4%
CRC#7	Metastasis	8%	17	553	27	272	65	292	0	210	5230	251	5%
CRC#8	Primary	14%	0	1193	469	614	3	349	0	689	2999	92	3%
CRC#9	Primary	8%	0	967	8	550	3	475	0	379	7973	204	2%
CRC#10	Primary	10%	0	733	<1	409	176	246	0	284	3463	124	3%
CRC#11	Metastasis	9%	1	1083	<1	481	300	567	0	410	5461	135	2%
CRC#12	Metastasis	4%	48	479	0	182	26	274	0	230	4937	106	2%
CRC#13	Metastasis	4%	3	356	5	135	289	163	0	168	3606	145	4%
CRC#14	Primary	2%	<1	260	7	139	305	119	0	143	5085	76	1%
CRC#15	Primary	17%	<1	1287	<1	715	51	530	0	419	6541	311	5%
CRC#16	Metastasis	5%	<1	477	44	237	89	197	0	219	3406	196	5%
CRC#17	Primary	17%	21	1067	49	539	42	382	0	323	3674	555	13%
CRC#18	Primary	14%	4	1078	81	550	33	370	<1	356	2016	262	12%
CRC#19	Primary	4%	3	288	<1	162	<1	120	0	135	4258	111	3%
CRC#20	Metastasis	22%	4	1580	19	754	10	584	<1	684	5604	391	7%
CRC#21	Metastasis	17%	10	1336	373	749	10	656	0	639	4894	432	8%
CRC#22	Primary	18%	0	2315	322	1081	32	908	0	1262	4671	1244	21%
CRC#23	Metastasis	8%	0	446	407	406	42	202	0	173	2360	155	6%
CRC#24	Primary	12%	0	981	5	581	13	508	0	331	4773	233	5%
CRC#25	Primary	5%	0	622	36	329	0	246	0	285	2643	68	3%
CRC#26	Primary	11%	0	1245	569	480	112	375	0	296	3607	237	6%
CRC#27	Primary	14%	4	1789	3	895	83	682	0	581	3101	891	22%
CRC#28	Carcinosis	5%	3	526	1	326	1	215	0	268	2545	29	1%
CRC#29	Primary	11%	5	1000	2	541	0	364	0	344	3172	391	11%
CRC#30	Primary	7%	0	753	11	332	22	282	0	258	2247	231	9%
CRC#31	Metastasis	10%	<1	629	1	294	29	241	0	216	2896	210	7%
CRC#32	Primary	16%	0	1073	304	556	28	357	0	469	1731	166	9%
CRC#33	Primary	7%	4	563	<1	277	7	202	0	218	1253	129	9%
CRC#34	Primary	13%	2	749	379	530	15	306	0	390	4293	157	4%
CRC#35	Primary	9%	0	958	3	484	9	329	0	318	2206	212	9%
CRC#36	Primary	21%	1	991	0	504	32	388	<1	436	3296	140	4%
CRC#37	Primary	19%	6	1978	16	840	10	391	0	668	2692	182	6%
CRC#38	Primary	8%	2	1114	8	446	2	320	0	367	1889	218	10%
CRC#39	Metastasis	12%	0	1156	478	523	40	366	0	418	4034	214	5%
CRC#40	Primary	10%	<1	547	94	356	49	199	0	242	1848	142	7%
CRC#41	Carcinosis	16%	0	1552	3	762	7	325	0	457	918	228	20%
CRC#42	Primary	31%	0	1786	<1	922	94	447	0	599	2710	493	15%
CRC#43	Primary	10%	0	1024	75	459	249	358	2	431	4126	272	6%
CRC#44	Carcinosis	15%	<1	938	159	565	1	285	0	364	2523	269	10%
CRC#45	Primary	12%	1654	807	512	388	9	215	1	332	969	124	11%
CRC#46	Primary	3%	<1	412	3	158	2	139	0	168	1865	61	3%
CRC#47	Metastasis	6%	0	521	2	252	<1	173	0	195	1662	68	4%
CRC#48	Carcinosis	10%	0	843	<1	417	0	252	0	252	1705	227	12%
CRC#49	Metastasis	6%	1	379	426	274	11	248	0	267	4587	149	3%
CRC#50	Metastasis	18%	31	1697	0	690	23	485	0	421	5271	299	5%
CRC#51	Primary	23%	0	1294	2	662	67	476	0	375	6660	583	8%
CRC#52	Primary	38%	14	3265	398	1126	640	736	0	836	7517	953	11%
CRC#53	Metastasis	19%	0	1657	0	566	15	430	0	430	4014	209	5%
***Median***		***10.6%***	***0.7***	***958***	***7***	***484***	***22***	***349***	***0***	***344***	***3463***	***210***	***6%***
**Ovarian carcinoma PDX**													
OVC#1	Metastasis	28%	42	2575	0	1498	89	1191	159	867	10390	459	4%
OVC#2	Metastasis	5%	4	565	99	350	2	439	34	259	6391	88	1%
OVC#3	Metastasis	21%	1	1427	304	809	69	406	<1	583	3133	710	18%
OVC#4	Primary	6%	26	709	9	474	0	259	4	272	1528	144	9%
OVC#5	Primary	7%	16	974	81	807	0	802	45	525	14226	95	1%
OVC#6	Primary	12%	3	2052	97	593	19	734	101	528	2628	427	14%
OVC#7	Primary	8%	0	762	4	470	0	270	32	278	6156	266	4%
OVC#8	Primary	3%	2	219	30	119	6	88.8	3	59.2	652	37	5%
OVC#9	Primary	8%	5	1795	2	674	3	518	5	372	2981	184	6%
OVC#10	Primary	4%	1	444	16	288	0	204	22	141	2812	52	2%
OVC#11	Primary	20%	24	1586	54	1036	0	482	2	648	2781	493	15%
OVC#12	Primary	13%	3	877	177	487	0	259	12	285	1720	127	7%
OVC#13	Primary	3%	17	550	207	263	2	196	<1	224	1134	16	1%
OVC#14	Primary	5%	0	332	<1	255	21	238	<1	164	19239	62	0%
***Median***		***7%***	***3.7***	***819***	***42***	***480***	***2***	***338***	***9***	***281***	***2896***	***136***	***5%***
**Glioblastoma PDX**													
GBM#1	Primary	8%	22	712	2051	457	378	559	8	186	18822	241	1%
GBM#2	Primary	13%	1	1351	1143	819	0	799	378	328	17084	296	2%
GBM#3	Primary	13%	1	2372	422	1184	0	1325	0	1237	8452	131	2%
GBM#4	Primary	5%	55	870	321	328	0	503	0	372	5923	78	1%
GBM#5	Primary	15%	0	2600	268	1389	28	1361	294	1413	15443	100	1%
***Median***		***13%***	***1.4***	***1351***	***422***	***819***	***0***	***799***	***8***	***372***	***15443***	***131***	***1%***
**Breast cancer carcinoma PDX**													
BC#1	Primary	2%	<1	222	204	113	3	89.4	2	80.7	637	114	15%
BC#2	Primary	8%	0	666	177	335	19	289	0	162	2997	310	9%
BC#3	Metastasis	10%	0	679	286	447	5	539	36	259	4961	334	6%
BC#4	Primary	15%	46	803	91	498	5	366	0	222	3547	447	11%
BC#5	Primary	1%	<1	116	0	61.9	33	89.1	4	31.9	15066	77	1%
BC#6	Metastasis	15%	2	1351	289	634	29	719	150	439	17360	357	2%
BC#7	Primary	22%	8	1908	442	887	0	1370	0	739	27659	365	1%
BC#8	Primary	6%	1	810	149	412	18	327	9	280	8360	160	2%
BC#9	Metastasis	10%	0	713	6	322	13	420	0	279	1020	294	22%
BC#10	Primary	6%	3	370	460	233	0	325	17	134	7447	154	2%
BC#11	Primary	6%	6	993	347	403	29	461	68	325	14282	256	2%
BC#12	Primary	8%	6	1005	466	543	28	664	3	391	25794	363	1%
BC#13	Primary	7%	0	575	92	256	8	266	15	189	6174	132	2%
BC#14	Primary	8%	654	745	45	413	0	279	2	253	3294	71	2%
BC#15	Metastasis	11%	4	912	50	461	1	311	0	286	3458	186	5%
BC#16	Primary	2%	0	199	188	94.6	4	73.6	2	69.7	610	100	14%
BC#17	Primary	4%	13	413	346	134	66	173	32	101	2131	197	8%
BC#18	Primary	10%	<1	1545	168	743	3	788	11	382	6550	167	2%
BC#19	Metastasis	16%	<1	2304	167	1188	5	1049	10	771	6004	280	4%
BC#20	Primary	17%	0	1967	340	959	1	709	20	634	11533	357	3%
BC#21	Primary	9%	0	730	334	332	2	476	91	202	8166	520	6%
BC#22	Primary	6%	0	598	451	222	10	334	90	124	5088	264	5%
BC#23	Primary	4%	0	377	331	179	2	195	19	83.3	1742	51	3%
BC#24	Primary	22%	1	1128	858	982	79	999	<1	495	21363	668	3%
BC#25	Primary	10%	0	1165	666	573	94	627	12	474	14542	244	2%
BC#26	Primary	10%	0	1446	429	572	0	685	230	510	5771	257	4%
BC#27	Primary	14%	2	880	4	452	94	415	<1	222	1505	299	17%
BC#28	Primary	5%	<1	182	91	113	7	119	6	80.3	1221	50	4%
BC#29	Primary	9%	<1	656	530	469	32	532	9	473	50360	255	1%
BC#30	Primary	7%	3	823	94	341	247	403	34	244	9097	373	4%
BC#31	Primary	4%	0	345	166	216	0	161	3	145	1085	79	7%
BC#32	Primary	7%	<1	629	13	276	4	237	19	194	1544	198	11%
BC#33	Primary	9%	<1	725	397	428	232	549	6	231	5414	144	3%
BC#34	Primary	14%	5	1061	245	557	0	457	0	308	3866	176	4%
BC#35	Primary	5%	2	484	103	358	3	506	50	185	23896	360	1%
BC#36	Primary	13%	0	1085	221	544	0	333	13	347	1153	231	17%
BC#37	Primary	4%	0	376	193	149	<1	144	13	120	1081	289	21%
BC#38	Primary	6%	2	776	90	415	8	326	12	281	4683	178	4%
BC#39	Primary	14%	0	961	5	731	83	691	4	606	4829	152	3%
***Median***		***7.9%***	***0.7***	***745***	***193***	***413***	***5***	***403***	***10***	***253***	***5088***	***244***	***4%***
**Cutaneous melanoma PDX**													
CM#1	Metastasis	11%	0	1725	2985	825	0	953	83	1026	10386	1401	12%
CM#2	Metastasis	7%	27	544	1391	426	768	360	0	184	10696	315	3%
CM#3	Metastasis	4%	1	282	257	180	0	122	1	155	599	102	14%
CM#4	Primary	20%	0	3306	784	1178	427	883	38	836	9188	718	7%
CM#5	Metastasis	8%	9	936	872	363	15	587	0	289	5590	201	3%
CM#6	Metastasis	3%	<1	342	648	196	6	188	5	236	960	23	2%
CM#7	Metastasis	1%	2	176	382	83.6	2	135	<1	84.3	5962	37	1%
CM#8	Primary	10%	9	4760	876	705	0	2230	5	841	16732	239	1%
CM#9	Metastasis	1%	0	118	284	61.6	20	125	14	73.6	3704	24	1%
CM#10	Primary	10%	0	876	756	300	0	248	279	285	387	126	25%
CM#11	Metastasis	8%	2	641	1102	427	0	355	1	309	8837	266	3%
CM#12	Primary	5%	0	530	112	186	0	440	0	423	683	26	4%
CM#13	Metastasis	2%	<1	243	145	101	<1	116	1	82.2	466	31	6%
***Median***		***7.1%***	***0.9***	***544***	***756***	***300***	***0.8***	***355***	***1***	***285***	***5590***	***126***	***3%***
**Renal cell carcinoma PDX**													
RCC#1	Primary	17%	0	1179	569	1002	0	680	2	473	19769	1513	7%
RCC#2	Primary	12%	0	3362	16	1929	0	1934	0	3043	27096	54	0%
RCC#3	Primary	27%	0	5431	411	2376	0	1866	0	1974	25792	211	1%
RCC#4	Metastasis	11%	120	2117	256	1430	4	1512	0	1337	13968	89	1%
RCC#5	Primary	16%	5	2906	33	1942	3	1624	0	2102	25817	87	0%
RCC#6	Primary	1%	1	341	2	125	2	157	47	119	609	13	2%
RCC#7	Primary	21%	0	768	549	1908	0	1292	0	1324	27232	157	1%
RCC#8	Metastasis	17%	1	842	410	778	0	466	0	286	1756	930	35%
RCC#9	Metastasis	13%	17	2024	230	1258	3	827	1	904	37839	55	0%
RCC#10	Primary	11%	0	2010	856	1359	0	1070	2	672	37217	83	0%
RCC#11	Primary	5%	2	597	907	350	0	487	0	253	5091	136	3%
RCC#12	Metastasis	14%	0	2546	257	1132	0	871	0	1040	16952	61	0%
RCC#13	Primary	21%	0	4963	38	3466	0	3281	0	3966	30645	155	1%
RCC#14	Primary	6%	330	1338	364	602	0	661	2	343	26952	52	0%
RCC#15	Primary	6%	77	565	1036	293	0	368	0	291	2210	59	3%
***Median***		***12.9%***	***1.2***	***2010***	***364***	***1258***	***0***	***871***	***0***	***904***	***25792***	***87***	***1%***
**Lung carcinoma PDX**													
***Small cell lung carcinoma***													
SCLC#1	Primary	8%	0	1030	3	387	0	250	0	196	419	43	9%
SCLC#2	Primary	3%	2	632	0	238	5	189	0	185	1300	52	4%
SCLC#3	Primary	4%	4	591	0	259	1	232	2	232	1117	49	4%
SCLC#4	Primary	3%	7	395	0	222	0	166	0	162	1498	46	3%
SCLC#5	Metastasis	2%	0	309	1	153	0	160	2	122	893	56	6%
SCLC#6	Primary	7%	2	670	9	221	471	208	72	192	954	86	8%
***Median***		***3.3%***	***1.7***	***612***	***0***	***230***	***1***	***198***	***1***	***189***	***1035***	***51***	***5%***
***Non small cell lung carcinoma***													
NSCLC#1	Primary	28%	3	1969	61	941	2	1145	14	637	18440	794	4%
NSCLC#2	Primary	8%	0	1270	0	611	0	511	335	639	5911	98	2%
NSCLC#3	Primary	22%	95	1590	31	1438	124	961	930	669	18346	429	2%
NSCLC#4	Primary	5%	2	686	5	339	4	212	59	221	875	85	9%
NSCLC#5	Primary	20%	3	1363	667	1387	184	896	3106	652	10612	688	6%
***Median***		***20%***	***2.7***	***1363***	***31***	***941***	***4***	***896***	***335***	***639***	***10612***	***429***	***4%***

Mouse cells encompass here a wide range of stromal cell types, including fibroblasts, inflammatory and immune cells, smooth muscle cells, and endothelial cells. We further focused on endothelial cells using expression of mouse *Pecam1*/*Cd31* and *Eng*/*Cd105* genes (hereinafter referred to as m*Cd31* and m*Cd105*, respectively) to evaluate their proportion within xenografts. *Vwf* gene encoding von Willebrand factor was also preliminary selected but not kept because of a lower expression rate in the mouse and human controls (Ct > 30, data not shown).

As expected, all samples, collected from large xenografts without necrotic centre, expressed m*Cd31* and m*Cd105* genes. Nevertheless, m*Cd31* and m*Cd105* mRNA levels widely varied between the samples (Table 1), but remained highly correlated to each other (*p* < 10^-7^; Table 2). Noteworthy, m*Cd31* and m*Cd105* expression levels were highly correlated with the proportion of mouse cells (Table 2), suggesting that the relative amount of endothelial cells remains stable within diverse stromal cell populations, whatever the density of stroma component and the cancer type.

**Table 2:** Relationships between mouse (m) and human (h) mRNA levels in the 150 human tumor xenografts

	***hCD31***	***mCd31***	***hCD105***	***mCd105***	***hVEGFR1***	***mVegfr1***	***hVEGFR2***	***mVegfr2***	***hVEGFA***	***mVegfa***
***mCd31***	0.025^1^									
	0.76^2^									
***hCD105***	0.043	0.121								
	0.60	0.14								
***mCd105***	0.040	0.928	0.189							
	0.63	**<0.0000001**	**0.02**							
***hVEGFR1***	0.065	0.022	-0.076	0.004						
	0.43	0.79	0.35	0.96						
***mVegfr1***	0.076	0.851	0.305	0.877	0.006					
	0.35	**<0.0000001**	**<0.0002**	**<0.0000001**	0.94					
***hVEGFR2***	0.010	-0.029	0.232	-0.036	-0.036	0.070				
	0.91	0.72	**<0.005**	0.66	0.66	0.40				
***mVegfr2***	0.003	0.912	0.173	0.919	-0.017	0.858	-0.090			
	0.98	**<0.0000001**	**<0.05**	**<0.0000001**	0.83	**<0.0000001**	0.27			
***hVEGFA***	0.095	0.477	0.319	0.563	0.090	0.726	0.131	0.517		
	0.25	**<0.0000001**	**<0.0002**	**<0.0000001**	0.27	**<0.0000001**	0.11	**<0.0000001**		
***mVegfa***	0.031	0.505	0.194	0.524	0.304	0.514	0.062	0.413	0.328	
	0.70	**<0.0000001**	**<0.05**	**<0.0000001**	**<0.0002**	**<0.0000001**	0.45	**<0.0000001**	**<0.00005**	
**% mouse cells**	-0.016	0.828	0.113	0.865	0.154	0.715	-0.145	0.797	0.364	0.666
	0.84	**<0.0000001**	0.17	**<0.0000001**	0.06	**<0.0000001**	0.08	**<0.0000001**	**<0.000005**	**<0.0000001**

Results, expressed as N-fold differences in target gene expression relative to the mouse and human *TBP* genes (both the mouse and human *TBP* transcripts) and termed “N*target*”, were determined as Ntarget = 2^∆Ctsample^ , where the ∆Ct value of the sample was determined by subtracting the average Ct value of target gene (human or mouse) from the average Ct value of ‘Total-*TBP’* gene). The N*target* values of the tumor samples were subsequently normalized such that the value for mRNA level was 1 when Ct=35. Target mRNA levels that were total absence or very low (Ct > 38) in tumor samples were scored ‘0’ for non expressed. As for calculation of % of mouse cells, specific mouse *Tbp* gene expression and the expression of both the mouse and the human *TBP* genes were studied by real-time qRT-PCR using the mouse *Tbp* as target gene and the ‘Total-*TBP’* as endogenous RNA control. Results, expressed as N-fold differences in specific mouse *Tbp* gene expression (using mouse *Tbp* primers) relative to the sum of the mouse and the human *TBP* gene expression (using ‘Total-*TBP’* primers), termed N*Mm-TBP*, are determined by theformula: N*Mm-TBP* = 2^DCtsample^. The DCt value of the sample is determined by subtracting the Ct value of the mouse *TBP* gene from the Ct value of the Total *TBP* gene. The N*Mm-TBP* values of the samples are subsequently normalized such that the median of N*Mm-TBP* values of 4 mouse tissues was 100. As *TBP* is a ubiquitously expressed housekeeping gene, showing similar expression in our human and mouse tissues (Ct=27 for 5 ng cDNA), the final result (normalized N*Mm-TBP* value) gives an estimate of the proportion of mouse cell content for a given xenograft. ^1^Spearman correlation coefficient, ^2^p value of Spearman rank correlation test, in bold when *p* is significant.

While numerous pro-angiogenic factors have been characterized, the VEGFA ligand has been identified as a predominant regulator of tumor angiogenesis and binds to VEGFR1 and VEGFR2 expressed on vascular endothelial cells. It mediates numerous changes within the tumor vasculature, including endothelial cell proliferation, migration, invasion, survival, chemotaxis of bone marrow-derived progenitor cells, vascular permeability and vasodilatation [1,2]. VEGFA expression by cancer cells is up-regulated by altered expression of oncogenes, a variety of growth factors and also hypoxia [2].

**Figure 1:**
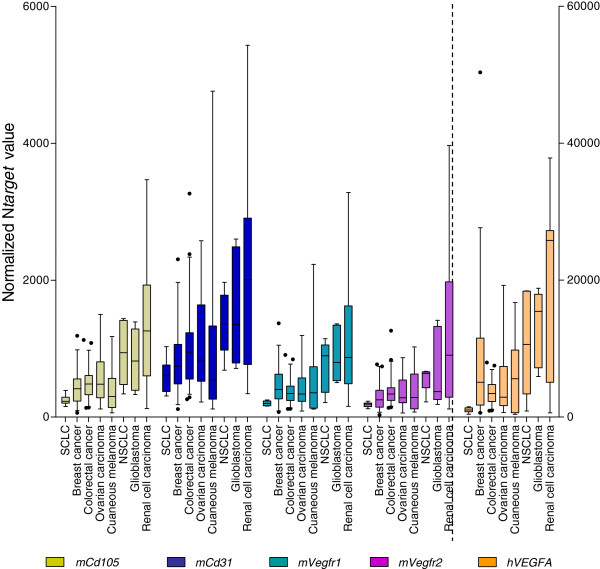
**Gene expression levels of mouse endothelial markers and *hVEGFA *in the 8 human tumor xenograft types.** Box-and-whisker diagrams showing the expression of mouse endothelial marker genes (*mCd31, mCd105, mVegfr1, mVegfr2)*, plot on left Y axis and *hVEGFA* gene plot on right Y axis. The box indicates the interquartile range, the centre horizontal line the median value and the black dots are outliers.

Unsurprisingly, we observed high levels of mouse F*lt1/Vegfr1*, mouse *Kdr/Vegfr2* (hereby denominated m*Vegfr1* and m*Vegfr2*) and human *VEGFA (hVEGFA)* transcripts, which correlated all with m*Cd31* and m*Cd105* RNA levels (Table 2). These strong positive correlations underline classical paracrine VEGFA-VEGFR1/2 signaling in tumorigenesis and crosstalk between the human ligand and mouse receptors. Expression of m*Vegfr1*, m*Vegfr2* and h*VEGFA* however varied widely in the different tumor types. RCC, glioblastoma and NSCLC xenografts showed transcript level median of these three genes at least 2 times higher than in the 5 other tumor xenograft types (Table 1, Figure 1). According to the expression level of m*Cd105*, m*Cd31*, m*Vegfr1*, m*Vegfr2* and h*VEGFA* (Figure 1)*,* the most angiogenic PDXs are then renal cell carcinoma, glioblastoma, and NSCLs, tumor types well-known to be the most angiogenic tumors in patients [11], underlying the interest of PDX models to mimic patient tumors.

Surprisingly, we observed also marked level of m*Vegfa* transcripts ranged from 50.7 (median in SCLC xenografts) to 429 (median in NSCLC xenografts). Individually, some xenografts showed more than 20% of the total *VEGFA* transcripts of mouse origin (Table 1). While VEGFA production by cancer cells is commonly reported, significant *VEGFA* expression has been also observed by fibroblasts and immune cells that surround and invade the tumor mass [12]. As reported by others [13], great attention has to be paid to mouse stromal VEGFA when anti-VEGF agents displaying specific human activity are tested in xenograft preclinical models.

Angiogenesis and vasculogenesis, mediated by angiogenic factors such as VEGFA are commonly accepted to support tumor vasculature. Vascular mimicry (ability of tumor cells to form functional vessel-like networks, devoid of endothelial cells) and cancer stem cell transdifferentiation into tumor endothelial cells are also two mechanisms recently reported in different tumors, including melanoma, breast, renal, ovarian cancer and glioblastoma [14-18] in which tumor cells directly participate in vascular channels. The presence of tumor-derived endothelial cells (TDECs) is usually investigated through the detection of CD31+ and CD105+ tumor cells [15-18]. TDEC cells are generally rare events and their identification needs highly sensitive methods (flow cytometry or confocal microscopy). Likewise, another approach to improving the detection of TDEC is to enhance the TDEC frequency by implanting into mice cancer stem cell enriched population. This prior enrichment could be done by culturing cells as tumor spheres [19,20] or by cell sorting for putative cancer stem cell markers [15,21]. Only one recent publication attempted to immunostain human CD31 directly in 3 human tumor xenografts, with no preliminary step of TDEC or CSC enrichment [22]. This study did not detect human CD31 and led the authors to conclude that endothelial cells in human hepatocellular carcinoma xenografts are of mouse rather than human origin, but did not allow them to absolutely exclude this possibility. Consequently, we apply in our PDX panel the real-time qRT-PCR method, known for its very high sensitivity, using human-specific *PECAM1*/*CD31* (*hCD31*) and *ENG/CD105* (*hCD105*) to gain more insight into TDECs.

**Figure 2:**
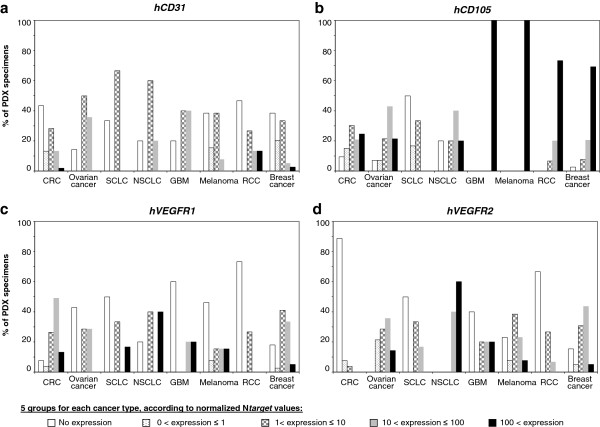
**Variations of human *hCD31 *(a), *hCD105 *(b), *hVEGFR1 *(c) and *hVEGFR2 *(d) gene expression within the 8 human tumor xenograft types.** Results are expressed for each cancer type as percent of PDX specimens showing normalized N*target* values in the following categories: no expression, 0 to 1, 1 to 10, 10 to 100 or more than 100.

Surprisingly, we detected h*CD31* and h*CD105* transcripts in all types of PDXs, suggesting that TDECs can exist in virtually all types of cancer. The possibility of human endothelial marker signals due to very rare remaining human stroma cells can not be ignored, although the whole human stroma in tumor xenografts is reported to be eventually replaced by stroma of mouse origin [8,23,24]. But depending upon the types, the range of expression of h*CD31* and h*CD105* transcripts largely varied (Figure 2a-b). All tested samples of cutaneous melananoma and GBM highly expressed h*CD105* gene (N*Hs*-ENG >100). Literature indeed reports a large expression of CD105, a member of the transforming growth factor beta receptor family, on normal and neoplastic cells of the melanocytic lineage, including melanoma cell lines, and an up-regulation in gene signature of aggressive cutaneous melanoma in patients [14]. Likewise, CD105 is highly expressed in glioblastoma but essentially absent in normal brain [21]. RCC xenografts displayed a great proportion of samples expressed high levels of h*CD31* or h*CD105*. These results fit with the literature that identified TDECs in patients mainly in glioblastoma and renal cancer [16,21]. By contrast, SCLCs show very low levels of both h*CD31* and h*CD105* mRNAs. A striking point is that h*CD31* and h*CD105* RNA levels did not correlate to each others (Table 2), even if their expression is analyzed for each cancer type (data not shown). It could be explained by different expression profiles for these 2 endothelial molecules: CD31 is considered as a pan-endothelial marker, whereas CD105 is a cell membrane glycoprotein predominantly expressed on cellular lineages within the vascular system, and over-expressed on proliferating endothelial cells [25]. These data underline that combination of markers is required to study the TDEC population.

Initially, VEGFRs were thought to be expressed only on endothelial cells, but these receptors may also be expressed on tumor cells and play a role in tumor resistance to existing therapies [5-7]. The present species-specific real-time qRT-PCR assays combined with our series of 150 PDXs represents a powerful tool to obtain further insight into autocrine and paracrine VEGFA-VEGR1/2 signaling in tumorigenesis. We indeed observed human VEGFR expression in xenografts with a profile that varied widely according to tumor types (Table 1, Figure 2c-d): High levels of h*VEGFR1* transcripts mainly observed in colon cancers and in NSCLCs; high levels of h*VEGFR2* transcripts in NSCLCs. Individually, 2 out of 5 NSCLC xenografts (i.e.: NSCLC#3 and #5) showed more h*VEGFR2* transcripts than m*Vegfr2* transcripts (Table 1). Conversely, SCLCs showed low levels of h*VEGFR1* and h*VEGFR2* transcripts and CRCs showed very low levels of h*VEGFR2* transcripts (Absence in 89% of the 53 CRC xenografts). These results identified NSCLC as an attractive cancer type for anti-VEGFR2 treatment. Small-molecule inhibitors as Sunitinib and Sorafenib are oral multikinase inhibitors, including VEGFR2 among their targets. The development of antibodies that can selectively block VEGFR2 could potentially result in improved potency or tolerability [3].

Whereras m*Vegfr1* and m*Vegfr2* expressions were extremely correlated to mouse endothelial markers (*p* < 10^-7^), human VEGFR profiles did not correlate highly with neither h*CD31* nor h*CD105*. Non exclusive hypotheses could explain this observation: i) human tumor cells expressing endothelial markers lead to VEGF- independent tumor vascularization with no expression of VEGFR1/2 [20]; ii) VEGFRs could be also expressed on carcinoma and participate to an essential autocrine/paracrine process for cancer cell proliferation and survival [1].

Collectively, VEGFA/VEGFR analyses suggest several autocrine and paracrine VEGFA-VEGFR1/2 signalings. In additional to the classical paracrine human tumoral VEGFA/mouse stromal VEGFR signalling, our data identified 3 others potential VEGFA-VEGFR signalings: a human cancer autocrine VEGFA/VEGFR signaling, an autocrine or paracrine mouse stromal VEGFA/VEGFR signaling, and a paracrine mouse stromal VEGFA/ human tumoral VEGFR signaling. It is noteworthy that the human cancer autocrine VEGFA/VEGFR signaling could occur intracellular, as well as by VEGFA secretion [6], limiting the quantity of extracellular VEGFA. Thus, VEGFR small-molecule inhibitors might be a more attractive therapy than VEGFA inhibitors which aim to sequestering free VEGFA.

To further investigate the potential value of species-specific PCR assays for *in vivo* evaluation of anti-angiogenesis therapy in PDX models, we analyzed in the same manner as described above, 2 NSCLC xenograft models after treatment with bevacizumab, a recombinant humanized monoclonal antibody to VEGF, approved for cancer therapy, including in NSCLC patients. These both models highly responded to one week-bevacizumab treatment in monotherapy: no tumor shrinkage but tumor stabilization throughout the experiment (Additional file 2: Figure S1).

As expected, the levels of m*Cd31,* m*Cd105,* m*Vegfr1* and m*Vegfr2* transcripts were significantly lower in the two bevacizumab-treated NSCLC xenografts as compared to matched non-treated xenografts (Table 3). Indeed, even if bevacizumab is able to bind and inhibit human VEGFA but unable to neutralize murine VEGFA, VEGFA in these 2 xenografts is produced by human cancer cells rather than by mouse stroma cells. It is noteworthy that one of the two xenografts (NSCLC#3) showed a significant upregulation of h*VEGFA* gene. More interestingly, the levels of h*CD31,* h*CD105,* h*VEGFR1* and h*VEGFR2* transcripts were not inferior in the two bevacizumab-treated NSCLC xenografts but on the contrary, h*CD31* was upregulated by 3 times (p < 0.05 for NSCLC#3) in both bevacizumab-treated xenografts. These data suggest that the mouse endothelial cells are more sensitive to anti-VEGFA therapy than human cells. Indeed, cancer cells are able to take advantage of autocrine intracellular VEGFA/VEGFR signalling [6] while bevacizumab is directed against free fraction of VEGFA. Furthermore, transdifferentiation of tumor cells into endothelial cells has been reported to be VEGF-independent but induced by HIF-1α [20]. Finally, bevacizumab induces hypoxia through mouse endothelial cells destruction, which may lead in turn to TDEC expansion. These latter results are of interest to apprehend molecular mechanisms of bevacizumab resistance.

**Table 3:** Target mRNA levels in 2 NSCLC xenografts after bevacizumab treatment

	**NSCLC#3**				**NSCLC#5**		
		**Control (n=5)**	**After bevacizumab reatment (n=5)**	***p-value***^**1**^	**Control (n=5)**	**After bevacizumab treatment (n=5)**	***p-value***^**1**^
***PECAM1/CD31*** **mRNA**	***Human***	18.1 (7.34-43.1)	57.6 (31.8-64.2)	**<0.05**	2.38 (0.00-9.21)	6.70 (2.41-17.1) NS	
	***Mouse***	863 (686-1790)	578 (483-847)	**<0.05**	2 334 (1 538-4 363)	856 (699-980)	**<0.05**
***ENG/CD105*** **mRNA**	***Human***	29.1 (3.59-47.2)	38.2 (15.1-71.4)	NS	57.64 (38.8-90.86)	57.50 (47.2 - 84.4)	NS
	***Mouse***	619 (580-1098)	414 (328-619)	**<0.05**	1 519 (1120-1813)	821 (610-860)	**<0.05**
***FLT1/VEGFR1*** **mRNA**	***Human***	59.6 (56.7-90.6)	88.9 (62.3-118)	NS	3.84 (0.00-24.8)	9.11 (3.87-20.3)	NS
	***Mouse***	589 (470-909)	274 (212-362)	**<0.05**	938 (633-1163)	305 (216-344)	**<0.05**
***KDR/VEGFR2*** **mRNA**	***Human***	507 (361-622)	545 (488-643)	NS	220 (140-274)	574 (213-834)	NS
	***Mouse***	466 (386-800)	204 (196-298)	**<0.05**	1 175 (698-1 211)	328 (316-349)	**<0.05**
***VEGFA*** **mRNA**	***Human***	20 503 (19162-24600)	32 160 (30 331-35 680)	**<0.05**	11 984 (5 368-13 961)	12 235 (7 088-14 042)	NS
	***Mouse***	160 (119-495)	307 (184-614)	NS	262 (170-680)	267 (240-360)	NS

Results are expressed as normalized N-fold differences in target gene expression relative to the ‘Total-TBP’ expression. These N*target* values of the tumor samples were normalized such that the value for the ’basal mRNA level‘ (Ct = 35) was ^1^Target mRNA levels that were total absence or very low (Ct > 38) in tumor samples were scored ‘0’ for non expressed.

Median and range in () are given for each gene in the different experimental conditions. ^1^Mann Whitney Test; NS, not significant; in bold, significant.

## Conclusions

The screening of a large panel of xenografts established from various tumor types is appropriate to identify the human tumor types that are likely to benefit from a new targeted therapy, and next to identify predictive biomarkers for the response to this targeted therapy. Human tumor xenografted models, closely mimicking clinical situations in terms of biological features and response to treatment [8], will also provide the necessary experimental conditions to evaluate fundamental issues in cancer, including characteristics of metastasis, angiogenesis, and tumor-stroma interactions. The present approach combining species-specific real-time qRT-PCR assays with a large cohort of patient-derived xenografts identified tumor endothelial cells in the all 8 tumor types tested and also revealed a complex pattern of both stroma and tumoral and both autocrine and paracrine VEGFA-VEGFR1/2 signalings. These both findings should be taken into account when evaluating molecular mechanisms of resistance to tumor anti-angiogenic strategies.

## Methods

### Patient-derived xenografts

Tumor xenografts have been established directly from patient tumors and were routinely passaged by subcutaneous engraftment in Crl:NU(Ico)-Foxn1^nu^ or CB17/Icr-Prkdc^scid^/IcrCrl [23,24,26-31] purchased from Charles River Laboratories (Les Arbresles, France), with protocol and animal housing in accordance with national regulation and international guidelines [32]. Xenografts were harvested here, after 5 to 12 passages into mice, when they reached around 2,000 mg in size.

Bevacizumab (Avastin, Roche) was given i.p. twice a week, one week, at 15 mg/kg in 0.9% NaCl. Omalizumab (Xolair, Novartis) is given as isotypic control. Lung carcinoma xenografts were transplanted into female 8-week-old Crl:NU(Ico)-Foxn1^nu^ mice. Mice with tumors of 60–200 mm^3^ were randomly assigned to control or treated groups. Tumor growth was evaluated by measurement of two perpendicular tumor diameters with a caliper twice a week. Individual tumor volumes were calculated: V = a × b^2^/2, a being the largest diameter, b the smallest. Mice were ethically sacrificed when the tumor volume reached 2 500 mm^3^ for control groups or at D29 and D50 after first injection of bevacizumab for NSCLC#2 and NCSCLC#3, respectively.

### Real-time RT-PCR

RNA extraction, cDNA synthesis and PCR conditions were previously described [33]. The precise amount and quality of total RNA in each reaction mix are both difficult to assess. Therefore, transcripts of the *TBP* gene encoding the TATA box-binding protein (a component of the DNA-binding protein complex TFIID) were quantified as an endogenous RNA control. The endogenous *TBP* control was selected due to the moderate prevalence of its transcripts and the absence of known *TBP* retropseudogenes (retropseudogenes lead to coamplification of contaminating genomic DNA and thus interfere with RT-PCR, despite the use of primers in separate exons) [9].

Quantitative values were obtained from the cycle number (Ct value) (Perkin-Elmer Applied Biosystems, Foster City, CA), according to the manufacturer’s manuals.

The gene primers (Additional file 1: Table S1) were chosen using the Oligo 6.0 program (National Biosciences, Plymouth, MN). The mouse and the human target genes primer pairs were selected to be unique when compared to the sequence of their respective orthologous gene. By contrast, a primer pair, referred as to ‘Total-*TBP’* primer pair*,* was selected to amplify both the mouse and the human *TBP* genes. dbEST and nr databases were scanned to confirm the total gene specificity of the nucleotide sequences chosen for the primers and the absence of single nucleotide polymorphisms. To avoid amplification of contaminating genomic DNA, one of the two primers was always placed at the junction between two exons. Agarose gel electrophoresis was used to verify the specificity of PCR amplicons. For each human-specific primer pair validation, we performed no-template control (NTC), no-human-reverse-transcriptase control (human RT negative), mouse-reverse-transcriptase control (mouse RT positive from a pool of normal and tumoral mouse RNAs extracted from various tissues types) assays, which produced negligible signals (Ct >40), suggesting that primer–dimer formation, genomic DNA contamination and cross species contamination effects were negligible. Same controls were realized for each mouse-specific primer pair.

### Statistical analysis

The distributions of mRNA levels were characterized by their median values and ranges. Relationships between mRNA levels of the different target genes were identified using nonparametric tests (GraphPad Prism 4.00, GraphPad Software, San Diego, CA).

## Abbreviations

CRC: Colorectal cancer; CSC: Cancer stem cell; GBM: Glioblastoma; NSCLC: Non small cell lung carcinoma; PDX: Patient-derived tumor xenograft; RCC: Renal cell carcinoma; SCLC: Small cell lung carcinoma; TDEC: Tumor-derived endothelial cell; mCd31: Mouse *Pecam1* gene encoding mouse CD31; mCd105: Mouse *Eng* gene encoding mouse CD105; mVegfr1: Mouse *Flt1* gene encoding mouse VEGFR1; mVegfr2: Mouse *Kdr* gene encoding mouse VEGFR2; hCD31: Human *PECAM1* gene encoding human CD31; hCD105: Human *ENG* gene encoding human CD105; hVEGFR1: Human *FLT1* gene encoding human VEGFR1; hVEGFR2: Human *KDR* gene encoding human VEGFR2.

## Competing interests

The authors declare no conflict of interest.

## Authors’ contributions

IB and VDM initiated the project and its design, contributed with data analysis and co-drafted the manuscript. SV contributed to the molecular gene studies and performed the statistical analysis. DV participated in project development, SR in sample preparation. RH participated in the molecular gene study. LDM, AD, FN, EA produced the PDX tissues. EM, SRR, DD participated in revision of the manuscript. All authors read and approved the final manuscript.

## Pre-publication history

The pre-publication history for this paper can be accessed here:

http://www.biomedcentral.com/1471-2407/14/178/prepub

## Supplementary Material

Additional file 1: Table S1 Sequences of oligonucleotides used. Click here for file

Additional file 2: Figure S1 Tumor growth curves of NSCLC#3 and NSCLC#5 xenografts as a function of time. Mice (at least 9 per group) were treated bevacizumab (•) at day 1 and 4; or not (o). Tumor volume was measured twice a week. Tumor growth was evaluated by plotting the mean of the RTV (relative tumor volume) ± SD per group over time after first treatment. Click here for file
