# Supplementary material for: Vasculature analysis of patient derived tumor xenografts using species-specific PCR assays: evidence of tumor endothelial cells and atypical VEGFA-VEGFR1/2 signalings
Source: BMC Cancer. 2014 Mar 13;14:178. doi: 10.1186/1471-2407-14-178 (PMC4007753; doi:10.1186/1471-2407-14-178)
Supplement: Additional file 1: Table S1 — Sequences of oligonucleotides used. [file 1471-2407-14-178-S1.pdf]

Supplementary Table 1 : Sequences of oligonucleotides used

Genes	Upper primer	Lower primer	Accession number
**Total-TBP**	5' TGC ACA GGA GCC AAG AGT GAA 3'	5' CAC ATC ACA GCT CCC CAC CA 3'	M_003194 + NM_01368
**Hs-TBP**	5' AGA ACA ACA GCC TGC CAC CTT AC 3'	5' GGG AGT CAT GGC ACC CTG AG 3'	NM_003194
**Mm-Tbp**	5' CCC TTG TAC CCT TCA CCA ATG AC 3'	5' TCA CGG TAG ATA CAA TAT TTT GAA GCT G 3'	NM_013684
**Hs-PECAM1**	5' CTG CTG ACC CTT CTG CTC TGT TC 3'	5' GGC AGG CTC TTC ATG TCA ACA CT 3'	NM_000442
**Mm-Pecam1**	5' GAC TCA CGC TGG TGC TCT ATG C 3'	5' TCA GTT GCT GCC CAT TCA TCA 3'	NM_008816
**Hs-ENG**	5' CAG CCT CAG CCC CAC AAG TCT 3'	5' GGG CCC ACA GGC TGA AGG T 3'	NM_000118
**Mm-ENG**	5' TAT AGC TTT GTA CCC ACA ACA GGT CT 3'	5' TGA GCT ACA CAG CCC TCG GA 3'	NM_007932
**Hs-VEGFR1**	5' ATC ATT CCG AAG CAA GGT GTG AC 3'	5' TCC TTC TAT TAT TGC CAT GCG CT 3'	NM_002019
**Mm-Vegfr1**	5' CCA CAA TCA CTC CAA AGA AAG GTA TG 3'	5' TCA ATT CTG TTT CCT AAG TTG CTG CT 3'	NM_010228
**Hs-VEGFR2**	5' TCA ACG TGT CAC TTT GTG CAA GAT AC 3'	5' CCA GGA AAT TCT GTT ACC ATC AGG A 3'	NM_002253
**Mm-Vegfr2**	5' TCA ATG TGT CTC TTT GCG CTA GGT AT 3'	5' GGG AGA GTA AAG CCT ATC TCG CTG T 3'	NM_010612
**Hs-VEGFA**	5' CTT GCC TTG CTG CTC TAC CTC C 3'	5' CAT CCA TGA ACT TCA CCA CTT CGT 3'	NM_003376
**Mm-Vegfa**	5' GCA CTG GAC CCT GGC TTT ACT 3'	5' ATG AAC TTG ATC ACT TCA TGG GAC T 3'	NM_009505
