# Supplementary figures and images for: Vasculature analysis of patient derived tumor xenografts using species-specific PCR assays: evidence of tumor endothelial cells and atypical VEGFA-VEGFR1/2 signalings
Source: BMC Cancer. 2014 Mar 13;14:178. doi: 10.1186/1471-2407-14-178 (PMC4007753; doi:10.1186/1471-2407-14-178)

## Supplementary Figure 1

**a**

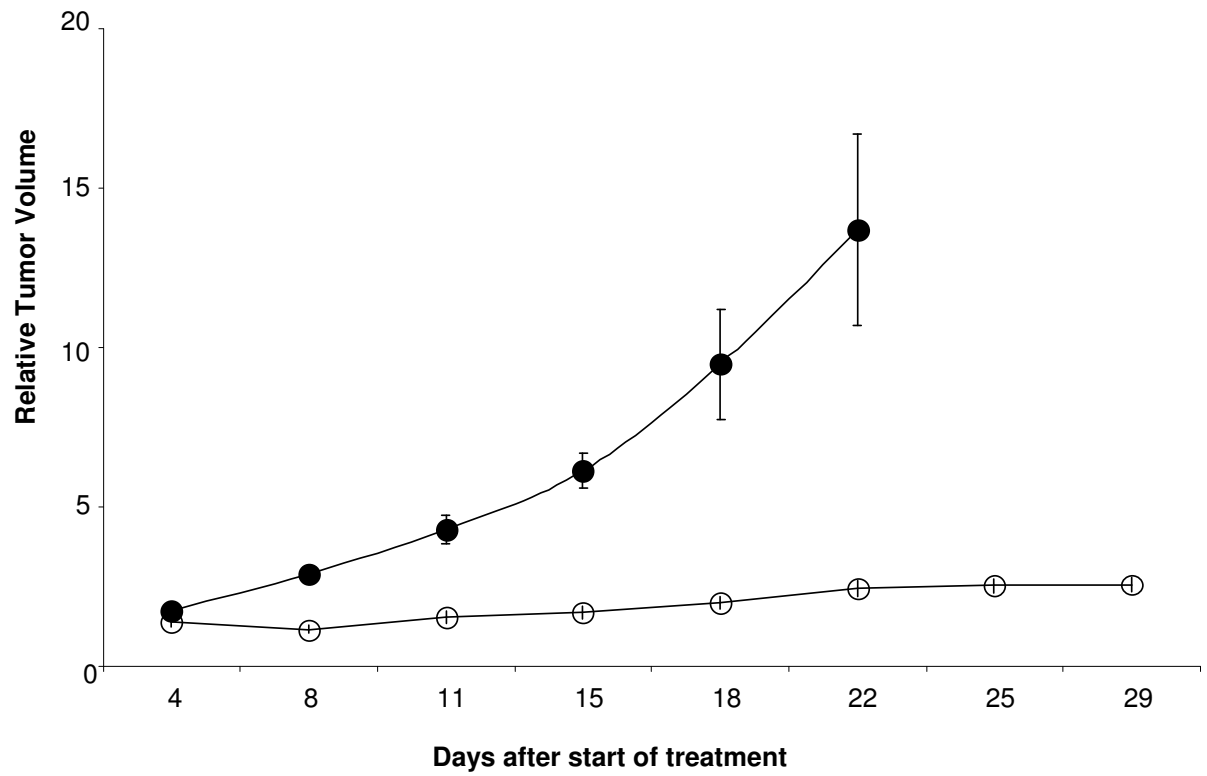

**b**

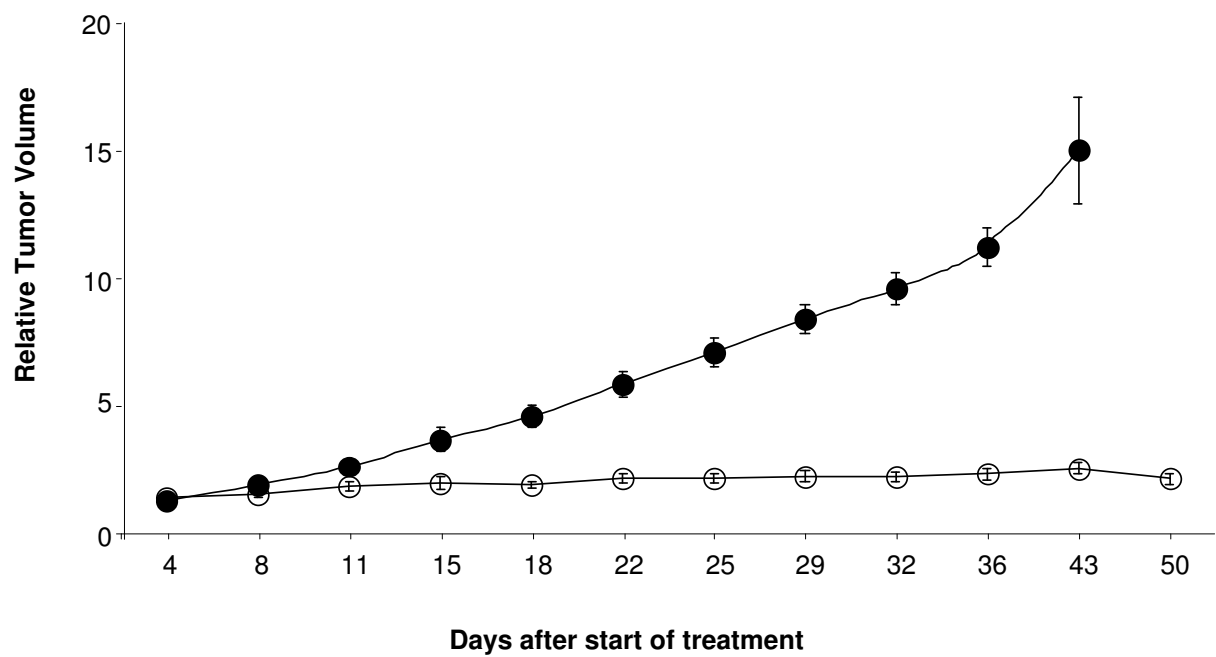

Supplement: Additional file 2: Figure S1 — Tumor growth curves of NSCLC#3 and NSCLC#5 xenografts as a function of time. Mice (at least 9 per group) were treated bevacizumab (•) at day 1 and 4; or not (o). Tumor volume was measured twice a week. Tumor growth was evaluated by plotting the mean of the RTV (relative tumor volume) ± SD per group over time after first treatment. [file 1471-2407-14-178-S2.pdf]
